# Chlor-Anodyne

**Published:** 1888-07

**Authors:** 


CHLOR-ANODYNE.—This preparation has justly acquired a high reputation as an anodyne. The formula as improved by Parke, Davis & Co., we have found most reliable. We have used it in doses of from 1 to 30 drops with very satisfactory results in cases when, on account of idiosyncracies or other causes, morphine could not be taken. In cases of nausea and vomiting it acts well, allaying the nausea and quieting the patient. It is well adapted to children, to whom we fiequently give it in broken doses with admirable results. In dysentery and mucus diarrhœa of children, after the action of a small dose of chalk and mercury or calomel, we have found that 15 drops added to a half glass of water, of which a teaspoonful may be given, makes an acceptable and efficient preparation, even where nausea exists. It should be given every hour until the evacuations are somewhat restrained, and then a dose after each action until relieved.

